# Adenomyosis and Uterine Fibroids: Two Distinct Yet Overlapping Clinical Entities

**DOI:** 10.3390/diagnostics16182968

**Published:** 2026-09-14

**Authors:** Giovanni Pecorella, Andrea Morciano, Mykhailo Medvediev, Radmila Sparic, İsmail Bıyık, Gercek Aydin, Giorgio Maria Baldini, Antonio Malvasi, Marta Stojković, Andrea Tinelli

**Affiliations:** 1 Department of Obstetrics and Gynecology, and CERICSAL (CEntro di RIcerca Clinico SALentino), “Veris delli Ponti Hospital”, Via Giuseppina Delli Ponti, 73020 Scorrano, LE, Italy; giovannipecorella2690@gmail.com; 2 Department of Gynaecology and Obstetrics, Pia Fondazione “Card. G. Panico”, 73039 Tricase, LE, Italy; drmorciano@gmail.com; 3 Department of Obstetrics and Gynecology, Dnipro State Medical University, 43100 Dnipro, Ukraine; medvedev.mv@gmail.com; 4 Faculty of Medicine, University of Belgrade, Dr Subotića 8, 11000 Belgrade, Serbia; radmila@rcub.bg.ac.rs (R.S.); mstojkovic037@gmail.com (M.S.); 5 Clinic for Gynecology and Obstetrics, University Clinical Centre of Serbia, Dr Koste Todorovića 26, 11000 Belgrade, Serbia; 6 Department of Obstetrics and Gynecology, Faculty of Medicine, Kütahya Health Sciences University, 43020 Kutahya, Turkey; dribiyik@hotmail.com; 7 Department of Obstetrics and Gynecology, Istanbul Health and Technology University Faculty of MedicineIstanbul, Sütlüce Mah. İmrahor Cad. No: 82, 34445 Istanbul, Turkey; op.drgercekaydin@gmail.com; 8 Türkiye and Eurofertil IVF Center, Hacı İlyas, Kıbrıs Şehitleri Cd. No:16/A, 16220 Bursa, Turkey; 9 Unit of Obstetrics and Gynecology, Department of Interdisciplinary Medicine (DIM), University of Bari “Aldo Moro”, Policlinico of Bari, 70124 Bari, BA, Italy; gbaldini97@gmail.com (G.M.B.); antoniomalvasi@gmail.com (A.M.)

**Keywords:** adenomyosis, uterine fibroids, transvaginal ultrasound, magnetic resonance imaging, differential diagnosis, fertility, personalized management, MUSA

## Abstract

Adenomyosis and uterine fibroids are among the most common benign uterine disorders in women of reproductive age and represent major causes of abnormal uterine bleeding, pelvic pain, and infertility. Despite their distinct histopathological features, they frequently coexist and share several hormonal and molecular mechanisms, making diagnosis and treatment challenging. This narrative review examines the current evidence on their epidemiology, pathophysiology, clinical presentation, imaging characteristics, reproductive impact, and therapeutic management, with particular attention to the role of imaging in the differential diagnosis and management of coexisting disease. A non-systematic narrative review of the literature was conducted using PubMed/MEDLINE and Scopus. Relevant original studies, systematic reviews, meta-analyses, consensus statements, and clinical guidelines addressing adenomyosis, uterine fibroids, imaging, fertility, and treatment were considered. Additional references were identified through manual screening of the bibliographies of the retrieved articles. Although adenomyosis and uterine fibroids differ in their biological origin and morphological appearance, they commonly present with overlapping symptoms, including heavy menstrual bleeding, dysmenorrhea, chronic pelvic pain, and reproductive dysfunction. Transvaginal ultrasound remains the first-line imaging modality, whereas magnetic resonance imaging is particularly valuable in complex cases, lesion characterization, and preoperative assessment. Accurate imaging phenotyping may help distinguish isolated from coexisting disease and support individualized medical, surgical, or minimally invasive treatment planning. A structured imaging-based approach integrated with clinical findings and reproductive goals may improve disease characterization and support individualized management of adenomyosis and uterine fibroids. Further standardization of imaging criteria and validation of emerging diagnostic technologies may improve patient stratification and treatment selection.

## 1. Introduction

Adenomyosis and uterine fibroids (leiomyomas) are among the most prevalent benign uterine disorders in women of reproductive age and represent a major cause of abnormal uterine bleeding, pelvic pain, and infertility in daily clinical practice [1,2]. They frequently present a diagnostic problem due to their great incidence and overlapping symptomatology, especially in non-specialized settings [3]. From a pathological standpoint, uterine fibroids are benign monoclonal tumors that originate from myometrial smooth muscle cells. Conversely, adenomyosis is characterized by the migration of ectopic endometrial glands and stroma within the myometrium, which is linked to hypertrophy of the surrounding smooth muscle tissue [4,5]. Both disorders are hormone-dependent and share molecular mechanisms, such as aberrant estrogen and progesterone signaling, chronic inflammation, and extracellular matrix remodeling, despite these different histological characteristics [6,7]. With the increasing use of high-resolution imaging techniques, reports of the common coexistence of fibroids and adenomyosis have increased due to these similar processes [8]. It may be challenging to distinguish the relative contributions of each ailment to the patient’s symptoms in certain situations, which has immediate consequences for clinical judgment and therapy choice [9]. The non-invasive diagnosis of these entities has been improved with the emergence of modern imaging techniques, especially magnetic resonance imaging (MRI) and transvaginal ultrasound with defined morphological criteria [10]. Adenomyosis has been linked to ultrasound characteristics such as myometrial heterogeneity, asymmetrical wall thickening, and myometrial cysts, while fibroids usually appear as distinct, spherical masses with varying echogenicity depending on their composition [11].

MRI further enhances diagnostic accuracy, especially in complex cases, by allowing detailed assessment of the junctional zone (JZ) and lesion characterization [12]. Adenomyosis and uterine fibroids should be viewed in this context as two distinct but sometimes overlapping clinical entities, with imaging criteria playing a major role in their distinction. Optimizing patient care and directing customized treatment plans require a thorough grasp of their diagnostic characteristics as well as their frequent coexistence. The aim of this narrative review is to compare adenomyosis and uterine fibroids from epidemiological, pathophysiological, clinical, imaging, and therapeutic perspectives. Particular emphasis is placed on their frequent coexistence, the role of imaging in the differential diagnosis, and the implications of accurate phenotyping for individualized clinical management and reproductive counseling (Table 1).

Table 1. Comparison of adenomyosis and uterine fibroids. Summary of the main epidemiological, pathogenetic, clinical, imaging, reproductive, and therapeutic characteristics of adenomyosis and uterine fibroids, highlighting their key similarities and differences. Treatment should be individualized according to symptoms, lesion location and morphology, disease extent, age, reproductive plans, other infertility factors, previous treatment, patient preferences, and local expertise. Evidence remains heterogeneous regarding fertility-preserving surgery, HIFU, UAE, and interventions before ART.

## 2. Materials and Methods

This article was designed as a non-systematic narrative review aimed at providing a clinically oriented overview of adenomyosis and uterine fibroids, with particular emphasis on their diagnostic overlap and imaging characteristics. PubMed/MEDLINE and Scopus were consulted to identify relevant English-language publications addressing epidemiology, pathophysiology, clinical presentation, imaging, reproductive outcomes, and management. Search terms included combinations of “adenomyosis”, “uterine fibroids”, “uterine leiomyoma”, “myoma”, “transvaginal ultrasound”, “magnetic resonance imaging”, “junctional zone”, “MUSA”, “abnormal uterine bleeding”, “pelvic pain”, “infertility”, “pregnancy outcomes”, “medical treatment”, and “surgical treatment”. Particular attention was given to systematic reviews, meta-analyses, consensus statements, clinical guidelines, and recent original studies, while seminal older publications were retained when relevant to the historical development of diagnostic concepts. Additional references were identified through manual screening of the bibliographies of selected articles. Given the non-systematic narrative design of this review, no predefined systematic-review protocol, formal study-selection process, risk-of-bias assessment, or quantitative data synthesis was applied.

## 3. Epidemiology and Clinical Burden

When evaluated by imaging or histological testing, the cumulative incidence rates of uterine fibroids, the most prevalent benign tumors of the female reproductive system, can reach 70–80% by the age of 50 [13]. Age, ethnicity, and diagnostic modality all affect their incidence; African-descendant communities and women having systematic imaging evaluation are routinely found to have greater rates [14,15]. Due to advancements in imaging techniques, especially transvaginal ultrasound and MRI, adenomyosis, which was previously thought to be a disorder that could only be recognized after hysterectomy, is now more frequently found in women of reproductive age [15]. According to recent imaging-based research, its prevalence ranges from 20% to 35%, albeit there is still variation because of variations in diagnostic standards and operator proficiency [16]. Reported coexistence rates vary substantially according to the population studied and the diagnostic reference standard. Rates approaching 30–50% have been described mainly in selected surgical or hysterectomy cohorts and should not be extrapolated to unselected outpatient, imaging-based, or fertility populations. TVUS- and MRI-based prevalence estimates may differ because of differences in diagnostic criteria, operator expertise, and patient selection [8,17]. When one ailment predominates on imaging, this overlap may result in an underestimate or incorrect classification of the other, adding to a more complicated clinical presentation [8,18]. The majority of clinical characteristics, such as irregular uterine bleeding, persistent pelvic discomfort, and reproductive dysfunction, which together lower quality of life and raise healthcare consumption, are also shared by the two entities [19].

Adenomyosis is becoming more widely acknowledged as a factor in chronic symptoms even following fibroid-targeted treatments, but uterine fibroids continue to be one of the most common reasons for hysterectomy globally. The absence of widely accepted criteria for adenomyosis, especially in ultrasound-based diagnosis, increases the diagnostic burden and may result in interobserver variability and underdiagnosis in ordinary clinical practice [20,21]. Typical leiomyomas are often readily recognizable because of their characteristic morphology; however, diagnostic uncertainty may arise in the presence of degeneration, multiple lesions, marked uterine distortion, focal adenomyosis, or coexisting disease [22]. In this regard, the diagnostic difficulty of these disorders is closely related to their epidemiological significance. To guarantee an accurate diagnosis and suitable treatment, better standardization of imaging criteria and a greater understanding of their frequent coexistence are crucial.

## 4. Pathophysiology: Distinct Origins, Shared Mechanisms

Although they originate from different biological processes, uterine fibroids and adenomyosis share a number of molecular and hormonal pathways that aid in their growth and development. Recurrent somatic mutations, most frequently involving the MED12 gene, have been discovered as important molecular drivers of leiomyoma development, which are benign monoclonal tumors derived from myometrial stem or progenitor cells [23]. The distinctive solid and well-circumscribed morphology seen on imaging is caused by these mutations, which encourage aberrant cellular proliferation and excessive extracellular matrix (ECM) deposition [24]. Adenomyosis, on the other hand, is thought to be a condition of the endometrial–myometrial interface, also known as the junctional zone. The two most commonly recognized pathogenetic mechanisms are metaplastic transformation of Müllerian remnants and invasion of the myometrium by the basalis endometrium [25]. These mechanisms, which result in localized hyperestrogenism, inflammation, and disruption of normal myometrial architecture, are believed to be mediated by recurrent tissue injury and repair (TIAR). Despite their different causes, both disorders are highly hormone-dependent, with progesterone and estrogen being essential for the development and maintenance of the disease [26]. Both conditions are hormone-dependent and share alterations in steroid-hormone signaling, inflammation, angiogenesis, fibrosis, and extracellular-matrix remodeling [27,28,29]. These common pathways may partly explain their frequent coexistence despite their distinct histopathological origins. Imaging results directly reflect these pathophysiological variations. Because of their confined growth and ECM-rich composition, fibroids usually appear as well-defined, encapsulated masses, while adenomyosis manifests as poorly defined, diffuse changes in the myometrium, reflecting its infiltrative nature and disruption of the JZ [12]. From a diagnostic standpoint, comprehending these pathways is crucial because they offer the biological foundation for deciphering imaging characteristics and differentiating between localized and diffuse uterine pathology. Furthermore, the frequent presence of various disorders and the possibility of diagnostic ambiguity in complex situations are explained by the overlap in molecular pathways.

## 5. Clinical Presentation and Symptom Overlap

The most frequent presenting symptoms of both uterine fibroids and adenomyosis are pelvic discomfort, dysmenorrhea, and abnormal uterine bleeding (AUB) [30]. Due to this overlap, it is frequently difficult to differentiate between the two entities using clinical examination alone, especially when high-quality imaging is lacking [31]. Heavy menstrual bleeding and bulk-related symptoms, like pelvic pressure, frequent urination, and constipation, are more commonly linked to uterine fibroids. These symptoms are mostly determined by the size, quantity, and anatomical position of the lesions [32]. Because of deformation of the uterine cavity, submucosal fibroids in particular are closely associated with heavy monthly bleeding and reproductive failure [33]. On the other hand, severe dysmenorrhea, persistent pelvic pain, and a diffusely swollen, painful uterus upon physical examination are more frequently associated with adenomyosis [34]. It is believed that increased uterine contractility, neuroangiogenesis, and local inflammatory processes inside the myometrium are linked to pain complaints in adenomyosis [35]. Because patients frequently arrive with a combination of bleeding and pain that cannot be clearly linked to a single pathology, the coexistence of both disorders complicates the interpretation of symptoms from a diagnostic standpoint [36]. According to multiple studies, women who have fibroids and adenomyosis together report worse quality of life and greater symptom severity levels than those who have either ailment alone [37]. Crucially, clinical assessment by itself has poor sensitivity and specificity for distinguishing between these entities, highlighting imaging’s crucial involvement in the diagnostic process [38]. Accurate morphological assessment with ultrasound and, when indicated, MRI supports the non-invasive clinical diagnosis and treatment planning, although histopathology remains the historical reference standard in uncertain cases [39]. Understanding the limitations of symptom-based diagnosis is crucial in this situation since depending solely on clinical presentation may result in incorrect diagnoses, treatment delays, or ineffective therapy approaches. Serum CA-125 levels may be elevated in women with adenomyosis and, in some studies, have been significantly higher than in women with leiomyomas. However, CA-125 lacks sufficient specificity and diagnostic accuracy to be used as a stand-alone biomarker and should be interpreted only as an adjunct to clinical and imaging findings [40].

## 6. Imaging and Diagnostic Challenges

Imaging is the primary means of accurately differentiating between uterine fibroids and adenomyosis because, in most situations, clinical examination is insufficient to do so [41]. Due to its accessibility, affordability, and good diagnostic performance when carried out by skilled operators, transvaginal ultrasonography (TVUS) is the first-line diagnostic modality [17,42]. The Morphological Uterus Sonographic Assessment (MUSA) group has proposed standardized sonographic criteria for adenomyosis, which include characteristics like myometrial cysts, hyperechoic islands, fan-shaped shadowing, asymmetrical myometrial thickening, and an irregular or interrupted JZ [11]. While no single sonographic feature is pathognomonic, myometrial cysts, hyperechoic islands, and echogenic subendometrial lines and buds are considered direct features of adenomyosis, whereas asymmetrical myometrial thickening, fan-shaped shadowing, translesional vascularity, and junctional zone abnormalities are considered indirect features [21]. On the other hand, uterine fibroids typically appear on ultrasound as distinct, round or oval masses with varying echogenicity; on Doppler imaging, they are frequently linked to edge shadowing and peripheral vascularization [22]. Even when there are several lesions, identification is made easier by the fibroids’ pseudocapsules and distinct separation from the surrounding myometrium [43]. Despite these distinguishing characteristics, focal adenomyosis (adenomyoma) can be difficult to diagnose because of its limited presentation and mass-like appearance, which can resemble fibroids [44]. However, adenomyomas typically lack a clearly defined capsule and exhibit unclear edges with internal variability, which reflects their infiltrative development pattern, in contrast to fibroids [45]. The differential diagnosis may become particularly challenging when leiomyomas undergo cystic, hemorrhagic, or hyaline degeneration, as their heterogeneous sonographic appearance and MRI signal characteristics may overlap with those of focal adenomyosis. Preservation of a well-defined margin or pseudocapsule and a predominantly peripheral vascular pattern favors leiomyoma, whereas an ill-defined infiltrative interface, myometrial cysts, and translesional vascularity support focal adenomyosis [21,45,46]. Nevertheless, substantial imaging overlap may persist, and unequivocal differentiation is not always possible. In complex or ambiguous instances, magnetic resonance imaging (MRI) is the preferred imaging technique due to its higher soft tissue contrast [42]. Available evidence suggests that TVUS and MRI may achieve broadly comparable diagnostic performance for adenomyosis when performed and interpreted by experienced operators, although reported accuracy varies according to the study population, reference standard, diagnostic criteria, and examiner expertise [17,47]. TVUS therefore remains the preferred first-line modality because of its accessibility and lower cost, whereas MRI is particularly valuable when ultrasound findings are inconclusive, when uterine anatomy is distorted by large or multiple leiomyomas, or when detailed characterization of focal disease and the junctional zone is required [17,42,47]. A maximal JZ thickness of 12 mm or greater has traditionally been considered suggestive of adenomyosis, although it should not be used as an isolated diagnostic criterion [48]. Junctional-zone thickness may be influenced by physiological and technical factors, including transient uterine contractions, menstrual-cycle phase, hormonal status, and image acquisition. Moreover, adenomyosis may occur without marked JZ thickening, whereas apparent JZ thickening may also be observed in the absence of adenomyosis. Accordingly, MRI assessment should integrate JZ morphology with other imaging signs rather than relying on a single thickness threshold [42,46]. Fibroids, in contrast, are typically well-circumscribed lesions with low signal intensity on T2-weighted images, although degeneration may substantially alter their appearance [46]. When evaluating the coexistence of fibroids and adenomyosis, MRI is especially useful because it enables accurate lesion mapping and assessment of the JZ, which is sometimes hidden on ultrasound in cases of large or numerous fibroids [46]. However, its higher cost and limited accessibility restrict its use as a first-line modality in routine clinical practice [42]. The absence of widely recognized imaging criteria and the considerable interobserver variability, especially with ultrasound-based assessment, are key limitations in the diagnosis of adenomyosis [47]. Although the revised MUSA terminology has improved standardization by distinguishing direct from indirect sonographic features, reproducibility remains variable across individual features and is influenced by examiner experience and image quality [20,21]. Consequently, standardized terminology does not eliminate operator dependence, and diagnostic performance obtained in expert ultrasound centers should be extrapolated cautiously to routine clinical practice. Structured reporting based on standardized terminology may nevertheless improve communication and facilitate comparison across examinations and studies. Diagnostic interpretation becomes particularly challenging when adenomyosis and leiomyomas coexist. Large or multiple leiomyomas may distort uterine architecture and limit assessment of the surrounding myometrium and junctional zone, whereas diffuse adenomyosis may alter myometrial echotexture and reduce lesion conspicuity. In these settings, MRI may provide additional anatomical information, although coexistence may remain difficult to characterize unequivocally even with multimodal imaging [42,46]. This diagnostic interaction has obvious therapeutic ramifications since inadequate diagnosis of one disease may result in poor treatment choices and chronic symptoms [49]. To increase diagnostic precision and reduce operator dependence, emerging techniques including MRI-based radiomics, ultrasound elastography, and three-dimensional ultrasound are being investigated [50,51,52]. These methods might make it possible to characterize myometrial pathology more objectively and distinguish between diffuse and focal disease patterns. Imaging is crucial in this changing environment for both diagnosis and management decision-making. Therefore, to adequately describe uterine pathology, especially in patients presenting with complex or overlapping disease, a thorough and consistent imaging assessment is necessary. The principal imaging features useful for the differential diagnosis of adenomyosis and uterine fibroids are summarized in Table 2.

Table 2. Imaging features of adenomyosis and uterine fibroids. Comparison of the principal pathological, morphological, ultrasonographic (TVUS), and magnetic resonance imaging (MRI) features of adenomyosis and uterine fibroids, highlighting the key imaging findings useful for differential diagnosis.

**Figure 1:**
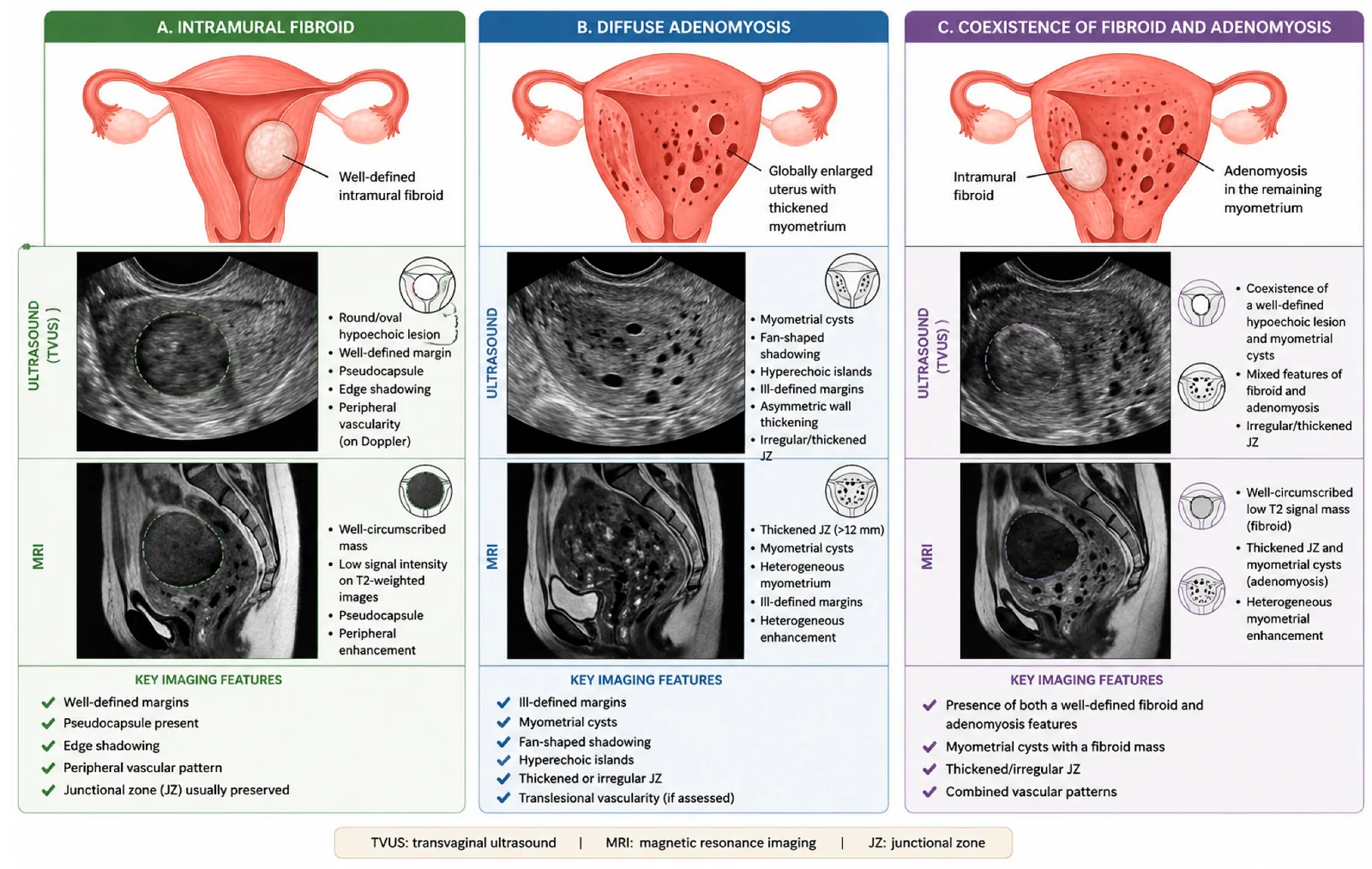
Imaging comparison of intramural uterine fibroid, diffuse adenomyosis, and coexisting disease. Schematic illustration of the typical morphological and imaging characteristics of (**A**) intramural uterine fibroid, (**B**) diffuse adenomyosis, and (**C**) their coexistence. For each condition, representative transvaginal ultrasound (TVUS) and magnetic resonance imaging (MRI) findings are summarized together with the key imaging features that support differential diagnosis. Fibroids typically appear as well-defined lesions surrounded by a pseudocapsule with peripheral vascularity, whereas adenomyosis is characterized by ill-defined myometrial changes, myometrial cysts, fan-shaped shadowing, and junctional zone abnormalities. In patients with coexisting disease, combined imaging features may coexist, highlighting the potential value of imaging phenotyping in individualized clinical assessment and management.

Overall, TVUS and MRI should be regarded as complementary rather than competing modalities. Neither technique provides a universally definitive diagnosis in every patient, particularly in focal disease or when adenomyosis and leiomyomas coexist. Diagnostic confidence depends on standardized morphological assessment, operator expertise, technical quality, and integration of imaging findings with the clinical context [17,21,42,47]. A schematic comparison of the characteristic ultrasound and MRI findings of isolated adenomyosis, uterine fibroids, and their coexistence is shown in Figure 1.

## 7. Impact of Adenomyosis and Uterine Fibroids on Fertility and Reproductive Outcomes

Adenomyosis and uterine fibroids have both been linked to decreased fertility and unfavorable reproductive outcomes; however, the underlying processes and amount of their effects vary according to lesion characteristics and disease severity [53]. Therefore, accurate diagnosis and phenotypic characterization using imaging may contribute to the assessment of infertile patients and support individualized reproductive counseling and treatment planning [54]. Because they deform the uterine cavity, change endometrial receptivity, and hinder embryo implantation, uterine fibroids—especially submucosal and large intramural lesions—are known to have a detrimental effect on fertility [33,55]. TVUS and MRI can be used to accurately measure their size, quantity, and proximity to the endometrial cavity [22]. To help with surgical decision-making, imaging-based classification algorithms have been developed to stratify fibroids depending on their influence on reproduction [56,57]. Adenomyosis has emerged as an independent factor affecting fertility, even in the absence of other uterine pathology [58]. Adenomyosis-related infertility has been linked to aberrant uterine peristalsis, chronic inflammation, delayed decidualization, and disruption of the JZ, all of which may impede early pregnancy development and embryo implantation [5]. Reduced implantation and conception rates have been linked to MRI-based evaluation of JZ thickness and disease degree, especially in individuals receiving assisted reproductive technologies (ART) [59]. The coexistence of adenomyosis and fibroids represents a particularly challenging clinical scenario, as their combined effects may further compromise reproductive outcomes [53]. In patients with coexisting disease, imaging may help characterize cavity distortion, lesion distribution, and the extent of adenomyosis, thereby contributing to individualized reproductive counseling and treatment planning. However, evidence that imaging-defined phenotypes themselves improve reproductive outcomes remains limited [33,53,60]. Adenomyosis is linked to lower clinical pregnancy rates, greater miscarriage rates, and lower live birth rates in ART cycles, particularly when diffuse or severe disease is present, according to several studies [60]. Similarly, fibroids that distort the uterine cavity are associated with impaired reproductive outcomes, including reduced implantation and pregnancy rates [33,55,61]. Preterm birth, placental abnormalities, fetal development limitation, and greater rates of cesarean delivery are among the obstetric problems associated with both diseases throughout pregnancy [62]. In certain situations, especially in women with large fibroids or severe adenomyosis, imaging follow-up may be necessary to track lesion evolution and evaluate potential concerns [63]. To maximize patient counseling and direct management choices in this situation, the integration of imaging results with clinical and reproductive data is crucial. In addition to improving characterization of disease burden, an accurate diagnostic evaluation may support more informed reproductive counseling and individualized treatment planning, although evidence that imaging-based strategies directly improve reproductive outcomes remains limited.

## 8. Imaging-Guided Decision-Making Algorithm

**Figure 2:**
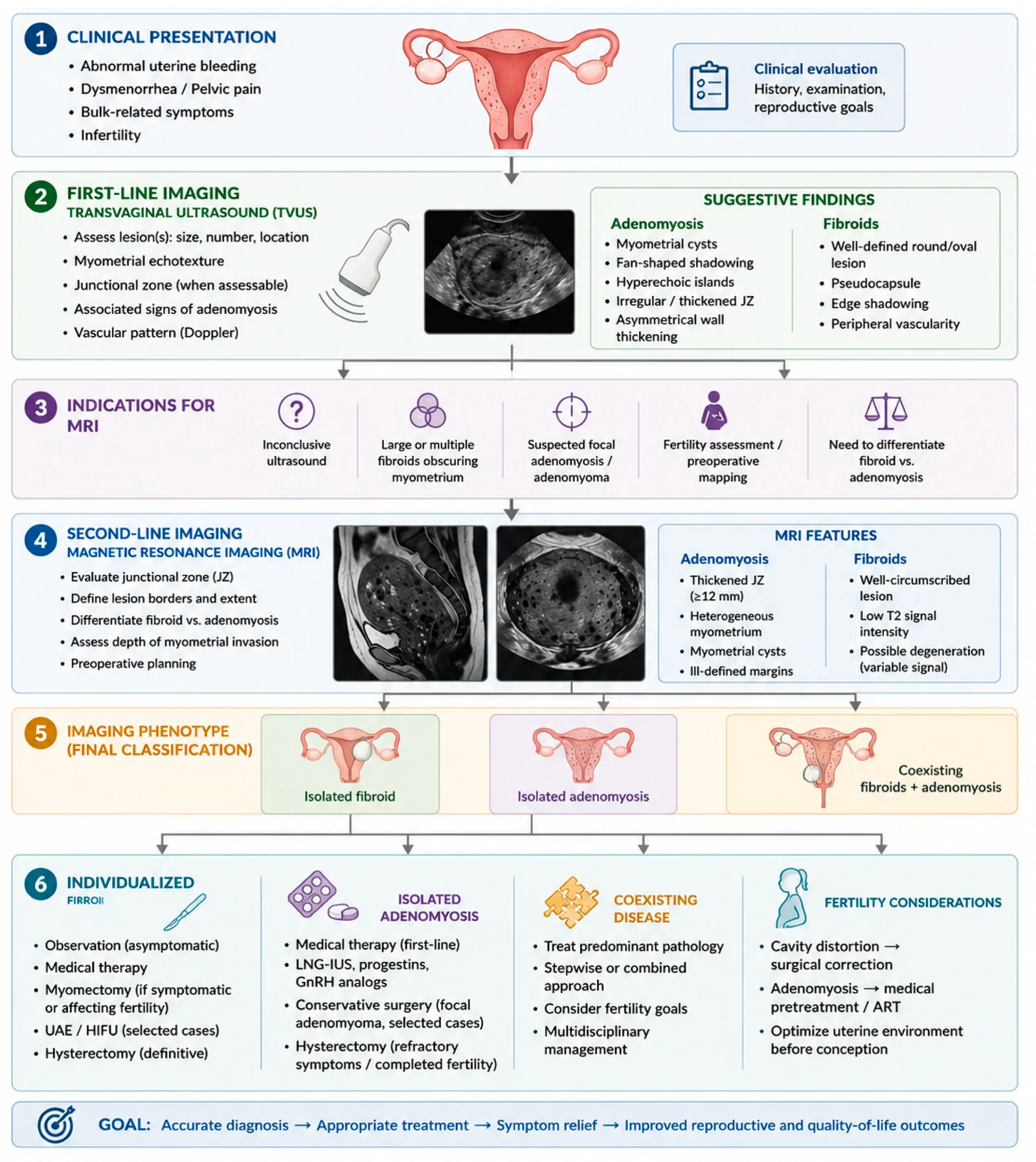
Authors’ proposed conceptual imaging-guided framework for adenomyosis and uterine fibroids. The framework integrates clinical presentation, first-line transvaginal ultrasound (TVUS), selective second-line magnetic resonance imaging (MRI), imaging phenotype, symptom profile, and reproductive goals. It represents a conceptual synthesis of the available evidence and should not be interpreted as a validated clinical guideline or management algorithm.

The diagnostic work-up for women who arrive with abnormal uterine bleeding, pelvic pain, infertility, or a combination of these symptoms should be organized around a stepwise imaging-based strategy that aims to differentiate between uterine fibroids, adenomyosis, and their coexistence [64,65]. Imaging provides important information from the initial diagnostic assessment to treatment planning, particularly because symptom patterns frequently overlap [66]. Due to its accessibility, affordability, and good diagnostic yield when carried out by skilled operators, transvaginal ultrasonography ought to be the initial imaging modality for all patients with suspected structural uterine pathology [11]. Lesion number, size, location, relationship with the endometrial cavity, myometrial echotexture, JZ irregularity when assessable, vascular pattern, and the presence of associated signs suggestive of adenomyosis, such as fan-shaped shadowing, myometrial cysts, hyperechoic islands, and asymmetrical wall thickening, should all be included in the initial ultrasound assessment [21,22]. Typical leiomyomas are often readily recognizable on ultrasound when they appear as well-defined round lesions with distinct margins, peripheral vascularization, and a characteristic pseudocapsule; however, diagnostic uncertainty may arise in the presence of degeneration, multiple lesions, marked uterine distortion, focal adenomyosis, or coexisting disease. Subsequent treatment can be customized based on FIGO type, symptom burden, reproductive plans, and uterine cavity distortion [56]. On the other hand, adenomyosis should be considered when imaging shows diffuse myometrial heterogeneity, an ill-defined lesion, or characteristics suggestive of JZ disruption, especially in women with severe dysmenorrhea, persistent pelvic pain, or reproductive failure [42]. Inconclusive ultrasound examinations, the presence of large or multiple fibroids obstructing the myometrium, suspected focal adenomyosis or adenomyoma, and fertility patients whose management may be affected by detailed phenotypic characterization may warrant a second-line MRI evaluation [12,59]. When ultrasound morphology is unclear, MRI helps distinguish focal adenomyosis from leiomyoma, enhances lesion mapping, and more clearly characterizes the JZ [46]. This is especially important when considering conservative surgery because infiltrative adenomyotic lesions and encapsulated fibroids differ significantly in terms of surgical technique, technical viability, and anticipated benefit [67,68]. Practically, imaging findings may inform therapeutic considerations across four main clinical scenarios. Lesion topography and symptom profile play a major role in the therapy of individuals with isolated fibroids. While asymptomatic lesions may be treated expectantly, submucosal fibroids and certain intramural fibroids impacting the cavity may warrant hysteroscopic or laparoscopic treatment [69,70]. Medical therapy may be considered for patients with diffuse adenomyosis, particularly when dysmenorrhea or heavy menstrual bleeding predominate and uterine preservation is desired [31]. Because localized adenomyosis, and especially adenomyoma, might resemble a fibroid morphologically but behave differently from a biological and surgical standpoint, care may necessitate a customized assessment [71]. In patients with coexisting fibroids and adenomyosis, imaging may help identify the predominant morphological features and clarify the potential contribution of each condition to the clinical presentation [42]. This distinction may also be relevant in reproductive medicine. Imaging may help characterize cavity distortion related to fibroids, the extent of adenomyosis, and a possible mixed phenotype as part of the overall infertility assessment [33]. Treatment planning should integrate these imaging findings with age, reproductive history, other infertility factors, previous treatment, patient preferences, and the planned ART strategy [61]. When several structural abnormalities coexist, or the clinical picture cannot be entirely explained by ultrasound results, MRI may be of considerable value in this situation [47]. The severity and extent of the disease should also be taken into account by an imaging-guided algorithm. Treatment planning for fibroids requires consistent reporting of size, number, FIGO classification, and cavity distortion [22]. The prognosis and treatment options for adenomyosis may be improved by differentiating between focal and diffuse disease, posterior versus anterior predominance, exterior versus inner myometrial involvement, and the existence of related endometriosis or fibroids [17]. Structured imaging reports can significantly enhance repeatability and interdisciplinary communication, even though there is currently no widely recognized severity categorization for adenomyosis [21,72]. In other situations, sophisticated imaging techniques may improve decision-making even further. Three-dimensional ultrasound may facilitate assessment of the coronal plane and junctional-zone morphology, while three-dimensional power Doppler permits quantitative evaluation of uterine vascularization. However, parameters such as vascularization index, flow index, vascularization-flow index, and uterine volume are not currently established as stand-alone diagnostic criteria for adenomyosis, and their incremental diagnostic value over standardized two-dimensional MUSA remains insufficiently validated [51]. These methods offer intriguing supplements for challenging cases and could help develop future precision-based diagnostic approaches, even though they are not yet included in standard clinical algorithms [73]. In general, an imaging-guided decision-making algorithm should be seen as an integrated therapeutic tool that connects morphology to symptoms, reproductive objectives, and available treatments rather than as a strictly diagnostic procedure. Accurate imaging phenotyping may reduce diagnostic uncertainty and support individualized management, particularly when fibroids and adenomyosis coexist. Overall, the proposed imaging-guided framework should be regarded as the authors’ conceptual approach integrating morphology, symptoms, reproductive goals, and available treatment options rather than as a validated clinical algorithm. Imaging phenotyping may support individualized clinical reasoning, but evidence demonstrating that such a pathway reduces treatment failure or improves patient-important outcomes remains limited. The authors propose a conceptual imaging-guided framework for adenomyosis and uterine fibroids in Figure 2.

## 9. Therapeutic Strategies: From Imaging Phenotype to Personalized Management

The treatment of uterine fibroids and adenomyosis has gradually moved toward an individualized approach in which treatment choice depends on symptoms, lesion location and morphology, disease extent, age, reproductive plans, other infertility factors, previous treatment, patient preferences, and local expertise [2]. However, imaging phenotype alone does not determine the optimal treatment and should be interpreted within the broader clinical context [2]. Treatment plans for patients with uterine fibroids are mostly based on the location of the lesion and the FIGO classification, as established by transvaginal ultrasonography or MRI [41]. Hysteroscopic or laparoscopic myomectomy is the most common surgical procedure used to treat submucosal fibroids and some intramural fibroids with cavity distortion, especially in women who are symptomatic or trying to conceive [55]. On the other hand, depending on the patient’s preferences and the course of their symptoms, asymptomatic fibroids or lesions without cavity involvement may be treated conservatively or with medication [6]. Treatment for adenomyosis should be individualized according to symptoms, disease extent and morphology, age, reproductive goals, previous treatment, and patient preferences. Medical therapy, including progestins, levonorgestrel-releasing intrauterine devices, or GnRH analogs, may be considered for symptom control in patients with diffuse adenomyosis [28]. Imaging contributes to the characterization of disease extent and morphology and may support individualized treatment planning together with clinical factors and patient preferences [54]. Adenomyoma, or focal adenomyosis, poses a unique therapeutic difficulty. These lesions may look like fibroids on imaging, but they have infiltrative edges and no obvious capsule, which greatly affects the viability and results of surgery [74]. Conservative surgery may be performed in certain situations, particularly in women who have significant symptoms or infertility; nevertheless, a thorough preoperative imaging evaluation is necessary to identify lesion borders, depth of invasion, and remaining myometrial thickness [75]. When fibroids and adenomyosis coexist, special care must be taken because treating one illness alone may not be sufficient to alleviate symptoms if the other condition is not identified [76]. Therefore, imaging may help characterize the relative contribution and extent of each disease phenotype as part of an individualized clinical assessment. For instance, myomectomy alone may not adequately address symptoms in patients with severe dysmenorrhea and diffuse adenomyosis, whereas surgical treatment of fibroids may be considered in selected patients with clinically relevant cavity distortion [69]. In certain cases, non-surgical, minimally invasive procedures including high-intensity focused ultrasound (HIFU) and uterine artery embolization (UAE) have become viable options. Lesion vascularity, size, and distribution are among the imaging features that may influence patient selection, technical feasibility, and treatment response [77]. UAE has shown promise in treating fibroids, but its effectiveness in treating adenomyosis is still debatable; greater results have been observed in cases of localized rather than generalized illness [78]. Similarly, HIFU requires careful imaging-based patient selection, although evidence regarding optimal selection criteria and comparative effectiveness across different disease phenotypes remains limited [79]. In reproductive medicine, cavity-distorting fibroids may warrant surgical consideration in selected patients, whereas hormonal pretreatment before ART has been investigated in women with adenomyosis. However, evidence supporting a uniform pretreatment strategy for adenomyosis remains heterogeneous, and treatment should be individualized according to symptoms, disease extent, previous reproductive history, and ART strategy [55,58,60,80]. Advanced ultrasound techniques and magnetic resonance imaging (MRI) can help stratify patients according to the severity of their diseases and direct customized solutions, including combined approaches. Overall, integrating imaging findings into clinical decision-making may support a more individualized approach to the management of uterine fibroids and adenomyosis. A phenotype-oriented approach based on morphological assessment may therefore support individualized treatment planning, although prospective evidence demonstrating superior patient-important outcomes compared with conventional management remains limited.

## 10. Future Perspectives: Artificial Intelligence, Radiomics and Biomarkers

It is anticipated that the combination of sophisticated imaging technologies with quantitative image analysis and artificial intelligence (AI) would greatly increase the diagnostic precision and repeatability in the assessment of uterine fibroids and adenomyosis [73]. Conventional imaging evaluation is sensitive to interobserver variability, notably in the diagnosis of adenomyosis, and is intrinsically operator-dependent, especially in ultrasound [17]. By providing automated feature detection and uniform interpretation of imaging data, AI-based solutions offer the ability to get around these restrictions [81]. A promising method for distinguishing between fibroids and adenomyosis and for assessing lesion heterogeneity beyond what is visually noticeable is radiomics, which entails the extraction of high-dimensional quantitative information from medical imaging [82]. According to preliminary research, radiomic signals obtained from MRI may enable more accurate categorization of uterine lesions, thereby enhancing diagnostic confidence in difficult circumstances such coexisting illness or adenomyoma [51]. Predicting the severity of an illness and how well a treatment would work has also been investigated using machine learning algorithms. Models that include imaging characteristics including lesion distribution, junctional zone thickness, and signal intensity patterns have demonstrated promise in predicting the severity of symptoms and reproductive outcomes in adenomyosis [9,59]. Finding non-invasive biomarkers outside of imaging is a field of ongoing research. Inflammatory mediators, angiogenic factors, and microRNAs have been investigated as potential biomarkers for improving disease characterization and monitoring [29]. However, none of these markers have yet demonstrated sufficient specificity and reproducibility to be implemented in routine clinical practice [29]. The integration of multimodal data, which combines imaging, clinical characteristics, and molecular profile into thorough prediction models, is probably going to be the foundation of the future diagnostic approach. Better phenotypic categorization, earlier diagnosis, and really individualized treatment plans could all be made possible by such an approach [83,84]. Despite these encouraging advancements, before these technologies can be widely used, more validation in extensive, prospective studies is needed. To guarantee repeatability and clinical applicability, standardization of imaging procedures, data collection, and analytical techniques will be crucial.

## 11. Conclusions

Given their shared pathophysiological pathways and frequent coexistence in clinical practice, adenomyosis and uterine fibroids should be recognized as two distinct but sometimes overlapping clinical entities. Clinical diagnosis is problematic due to their varied presentation and overlapping symptomatology, underscoring the crucial role imaging plays in precise disease characterization. Complementary techniques that enable in-depth morphological evaluation, distinguish between focal and diffuse disease, and detect coexisting pathology are transvaginal ultrasonography and magnetic resonance imaging. Therefore, an imaging-based strategy is central to disease characterization and may support individualized treatment planning, although direct evidence that imaging-based phenotyping improves patient-important outcomes remains limited. The need for a thorough and standardized diagnostic framework that can integrate various imaging aspects with clinical and reproductive data is highlighted by the growing acknowledgment of the coexistence phenotype. In this regard, cutting-edge technologies like biomarker research, radiomics, and artificial intelligence have the potential to enhance diagnostic accuracy and allow for customized treatment plans. Standardizing diagnostic criteria, validating sophisticated imaging technologies, and incorporating multimodal data into models that can be used in clinical settings should be the main goals of future initiatives. These advancements may contribute to improving the diagnostic process and management of women with these common uterine disorders.

## Figures and Tables

**Table 1:** Comparison between adenomyosis and uterine fibroids.

Feature	Adenomyosis	Uterine Fibroids
Origin	Ectopic endometrial glands and stroma within the myometrium	Benign monoclonal smooth muscle tumor
Pathogenesis	Endometrial invasion, TIAR hypothesis, Müllerian metaplasia	MED12 mutations, HMGA2 alterations, extracellular matrix accumulation
Typical age	35–50 years	30–50 years
Hormonal dependence	Estrogen- and progesterone-dependent	Estrogen- and progesterone-dependent
Main symptoms	Dysmenorrhea, heavy menstrual bleeding, chronic pelvic pain	Heavy menstrual bleeding, bulk-related symptoms, infertility
Uterine morphology	Diffuse or focal myometrial enlargement	Well-defined focal mass(es)
TVUS findings	Myometrial cysts, fan-shaped shadowing, hyperechoic islands, irregular junctional zone, asymmetrical wall thickening	Round hypoechoic lesion, edge shadowing, pseudocapsule, peripheral vascularity
MRI findings	Thickened junctional zone, heterogeneous myometrium, ill-defined lesions	Well-circumscribed low-T2 signal lesions
Fertility impact	Impaired implantation, abnormal uterine peristalsis	Cavity distortion, impaired implantation
Commonly used medical options	LNG-IUS, progestins, GnRH agonists/antagonists	GnRH antagonists, SPRMs * (where available), hormonal therapy
Potential procedural or surgical options	Conservative adenomyomectomy (selected cases), hysterectomy	Myomectomy or hysterectomy
Typical imaging challenge	Focal adenomyosis (adenomyoma) mimicking leiomyoma	Degenerated leiomyoma mimicking adenomyosis

* stands for Selective progesterone receptor modulators.

**Table 2:** Imaging features of adenomyosis and uterine fibroids.

	Adenomyosis	Uterine Fibroids
**Pathology**	Endometrial glands and stroma within the myometrium	Benign smooth muscle neoplasm
**Morphology**	Diffuse or infiltrative	Well-circumscribed mass
**TVUS**	Myometrial cysts, fan-shaped shadowing, hyperechoic islands, irregular JZ	Round lesion, edge shadowing, pseudocapsule, peripheral vascularity
**MRI**	Thickened JZ, heterogeneous myometrium, myometrial cysts	Well-defined low-T2 lesion
**Vascularity**	Translesional	Peripheral
**Main diagnostic pitfall**	Focal adenomyosis (adenomyoma)	Degenerated leiomyoma

## Data Availability

No new data were created or analyzed in this study. Data sharing is not applicable to this article.

## Abbreviations

The following abbreviations are used in this manuscript:

MRI	Magnetic resonance imaging
ECM	Extracellular matrix
TIAR	Tissue injury and repair
AUB	Abnormal uterine bleeding
JZ	Junctional Zone
TVUS	transvaginal ultrasonography
MUSA	Morphological Uterus Sonographic Assessment
FIGO	International Federation of Gynecology and Obstetrics
ART	assisted reproductive technologies
HIFU	high-intensity focused ultrasound
UAE	uterine artery embolization
AI	artificial intelligence
